# Implant-Related Infection in the Tibia: Surgical Revision Strategy with Vancomycin Cement

**DOI:** 10.1155/2014/124864

**Published:** 2014-11-05

**Authors:** Yong-Qing Xu, Yue-Liang Zhu, Xin-Yv Fan, Tao Jin, Yang Li, Xiao-Qing He

**Affiliations:** Orthopaedic Department, Kunming General Hospital of Chengdu Military Region, No. 212, Daguan Road, Kunming, Yunnan 650032, China

## Abstract

The development of a deep wound infection in the presence of internal hardware presents a clinical dilemma. The purpose of the present study was to evaluate the treatment outcomes of vancomycin cement with other advances of surgical techniques for implant-related infection (IRI) in the tibia. This study included 217 consecutive patients who had sustained IRI of the tibia. Of them, 152 patients had soft tissue defects and the internal hardware was exposed. Repeated debridement and negative pressure assisted closure were used. All the infected internal hardware was removed. External fixations and flaps were used. Custom-made vancomycin cement was inserted into the dead space of the wounds and left in site for a month. The follow-up was from 12 months to 108 months, averaging 37.5 months. For all the 217 patients, the general osteomyelitis healing rate and bone union rate were 86.6% and 97.2%, respectively. This study shows high rates of healing of IRI in the tibia if the new advances of surgery could be effectively combined into the treatment strategy with vancomycin cement as an important treatment.

## 1. Introduction

The incidence of placement of osteosynthetic materials has grown worldwide. In traumatic orthopaedic surgery, the treatment of the tibia fractures with plates or intramedullary nails is widely accepted. However, the postoperative implant-related infection (IRI) may occur and leads to serious outcomes. It remains challenging and expensive to treat, despite advances in antibiotics and new operative techniques. The traditional management includes irrigation and debridement, obliteration of dead space, intravenous antibiotics, and removal of the hardware [1–4]. These conventional therapies may not be sufficient for the infections. In the past fifteen years, we have been trying additional methods including self-made vancomycin cement, negative pressure assisted closure, external fixators, and new flap choices for the treatment. We find that these methods could be effectively combined and work together for IRI in the tibia.

## 2. Materials and Methods

This retrospective study obtained approval of the local ethical review committee and included 217 consecutive patients with implant-related infection of the tibia from 1998 to 2013. Mean patient age was 35.6 years (19–55 years). These patients all come from level-I trauma centers. According to their hospital charts, there were 52 closed fractures and 165 open fractures which were subdivided by Gustilo type I (*n* = 83), type II (*n* = 47), type III A (*n* = 21), and type III B (*n* = 14). After primary surgeries, 152 patients had soft tissue defects and the internal hardware was exposed. The soft tissue defects ranged from 4 × 3 cm to 30 × 12 cm. The other 65 patients had simple osteomyelitis with one or several sinuses. The internal implants included plates (*n* = 157) and intramedullary nails (*n* = 60). Those with bone defects longer than 1 cm were not included in this study. Any patients with a loss of follow-up were not included in this study.

The general health of the patient comes first in our treatment strategy. Attention should be paid to good nutrition, to a smoking cessation program, and to control of specific diseases such as diabetes. Thus, an attempt is made to improve the nutritional, medical, and vascular status of the patient and to provide optimal treatment of any underlying disease.

Antibiotic therapy was initiated with a cephalosporin. After culture specimens are obtained by means of a bone biopsy or during debridement, a parenteral antimicrobial regimen is begun to cover the clinically suspected pathogens. Once the organism is identified, the treatment may be modified according to the sensitivity of the isolated microorganisms. It lasted four to six weeks, dated from the last major debridement. If the initial medical management fails and the patient is clinically compromised by a recurrent infection, bone and soft tissue debridement is necessary in conjunction with another four-week course of antibiotics.

Debridement of bone was done until punctate bleeding was noted when the tourniquet is released. If in any doubt, we would extend the resect area of the bone and other tissues. We usually use vacuum sealing drainage system for the continuous wound drainage before the definitive treatments.

All the infected internal hardware, including the plate-and-screw constructs and intramedullary nails, was routinely removed. External fixators, including monolateral, hybrid, and ring structures, were used for the tibia fixation until the bone healed.

Adequate debridement always leaves a dead space. We fill this dead space with the vancomycin cement. The cement (Refobacin Bone Cement R, Biomet) and vancomycin were mixed with a ratio of 10 g : 0.5 g. The diameter of the mixed cement bar was from 0.5 cm to 0.8 cm according to the size of dead space. The cement bar was inserted into the intramedullary cavity without dead space. We would leave the vancomycin cement in the intramedullary cavity for a month and then pull it out with a rate of 1 cm each day. During this period, no irrigation of the wound was given.

One hundred and fifty-two patients had soft tissue defects and the size ranged from 4 × 3 cm to 30 × 12 cm. The flaps we used were sural flaps (*n* = 50), pedicled gastrocnemius flap (*n* = 26), pedicled fibular perforator flaps (*n* = 45), free anterolateral thigh perforator flaps (*n* = 23), free DIEP flaps (*n* = 3), and free thoracodorsal artery perforator flaps (*n* = 5).

**Figure 1:**
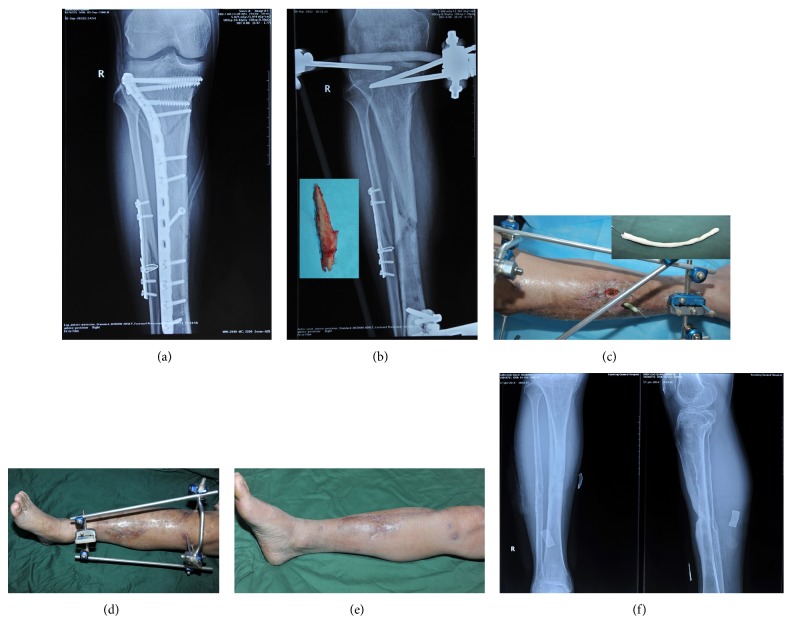
A patient sustained a Gustilo III A fracture in the tibia and received a plate-and-screw fixation. Two weeks later, there was infection on the wound. After debridement, persisting irrigation, and temporary reservation of the hardware for another two weeks, there were no signs of healing (a). The plate of the tibia was removed and replaced by assembled external fixators. A dead bone could be seen on the X-film and was later removed (b). After sufficient debridement, a custom-made vancomycin cement rod wias was inserted into the sinuses and the intramedullary cavity (c). Six months after the surgery, the sinuses and the osteomyelitis healed (d). Eighteen months after our surgery, both the wound and the bone healed uneventfully ((e), (f)).

**Figure 2:**
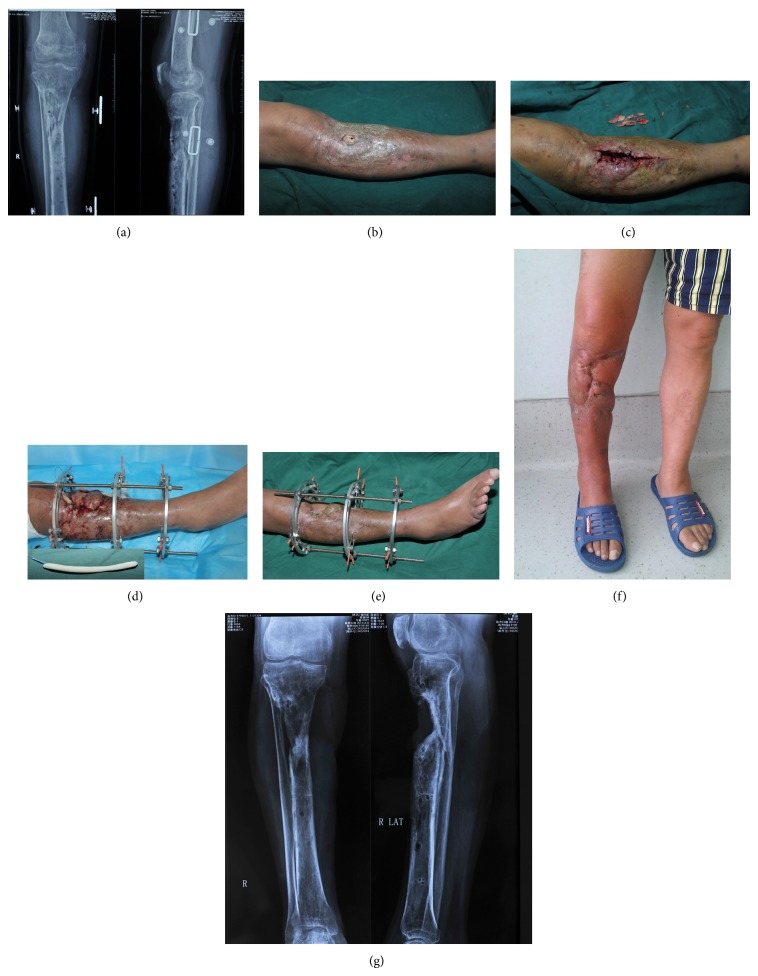
A 35-year-old male with a proximal diaphyseal fracture had received a plate fixation and later was removed due to IRI (a). A sinus existed in the proximal tibia and the bone was exposed (b). During the debridement, all the dead bones were removed (c). The wound was closed with the medial gastrocnemius flap and vancomycin cement rod in site (d). Two months later, the wound healed (e). At two-year follow-up, the wound and the bone healed well. The patient could walk normally ((f), (g)).

Each patient, with or without soft tissue defects, received the combination of the hereby mentioned techniques (Figures 1 and 2).

## 3. Results

Microbiological results showed that 87.1% (*n* = 189) of patients had positive intraoperative bacterial detection within the bone tissue, confirming osteomyelitis. The follow-up was from 12 months to 108 months, averaging 37.5 months. For all the 217 patients, the general osteomyelitis healing rate and bone union rate were 86.6% and 97.2%, respectively (Table 1). The healing time of the osteomyelitis ranged from 2 to 14 months, averaging 3 months. The bone union time ranged from 3.5 to 20 months, averaging 14 months. Nineteen patients of the osteomyelitis failed to heal after all the aforementioned treatments. Among them, 6 patients (4 had Gustilo IIIb and 2 had Gustilo II) had finally the nonunion of the tibia and two of them received amputations (1 had Gustilo IIIb and 1 had Gustilo II). The other 13 patients, including the four with nonunion, were eventually treated with distraction osteogenesis. Of all the 14 fractures with Gustilo IIIb, the rate of the bone union and osteomyelitis healing is 71.4% (10 among 14).

## 4. Discussions

Posttraumatic and postoperative osteomyelitis is one subtype of osteomyelitis with increasing prevalence and extends to approximately 80% of all cases [5]. Its pathophysiology of bacteria inoculation could be provoked by a direct contamination during trauma—particularly in open fractures or during associated fracture fixation surgery [2]. The presence of a big internal hardware increases the possibility of the infection. In the past forty years, the pathogenesis of the IRF has almost been clarified, and many factors accounting for the persistence of infection had been identified. One important pathophysiologic factor with increasing attention is the invincible bacterial biofilm attached to fixation implants [6]. Further risk factors for bacterial soft tissue, bone, and IRI include extrinsic factors such as fracture severity and soft tissue damage [3]. Accordingly, the rate of infection varies from 1 to 5% for open reduction and internal fixation and is increased to much higher rates in open, comminuted fractures [7–10]. In our series, a large part of the patients (*n* = 165) had open fractures which indicated that fracture severity and soft tissue damage may be two important parameters to predict the clinical outcome once IRI has occurred. However, the detailed pathophysiologic route of bacterial inoculation remains unclear. Osteomyelitis could be initiated by bacterial inoculation during trauma or during fracture fixation, respectively. Both routes may contribute to the bone infection.

Failure occurs mostly as a result of emergency of resistant strains or inadequate surgical debridement. And we believe that most treatment failures are probably due to a lack of adequate surgical debridement rather than inadequate antibiotic efficacy. The removal of the dead and infected tissue should be as thoroughly as the dissection of a malignant tumor. Without adequate debridement, the failure rate is high regardless of the duration of therapy. Even when all necrotic tissues have been adequately debrided, the remaining bed of tissue must be considered contaminated with the responsible pathogen or pathogens. Therefor, it is important to treat the patient with antibiotics for at least four weeks.

An additional option that may aid healing of soft tissue wounds and termination of IRI is the vacuum assisted closure system. This system began from 1992 and 1993 [11, 12] and now became a worldwide technique for the surgeons in traumatic orthopaedic [13]. To our knowledge, this device helped a lot to increase the rate of granulation tissue formation and the success rate of flap coverage.

A mechanical stability is important for an adequate bone healing [14]. Therefore, some authors consider at least temporary implant retention and suggest eradication of infection once bone healing is completed [15]. On the other hand, the maintenance of the biofilm-covered implants may contribute as additional risk factor for persisting or reoccurring chronic osteomyelitis [16]. In the past fifteen years, we have made many attempts to maintain the internal implants during the treatment of IRI and found that the success rate was very low. The external fixators, if properly used, could maintain the fracture stability well. Now we recommend a rather aggressive surgical strategy with radical debridement and removal of all internal implants.

Polymethylmethacrylate (PMMA) cement is considered the standard vehicle for local antibiotic delivery [17–21]. Antibiotic-impregnated acrylic beads may be used to sterilize and temporarily maintain a dead space. The beads are usually removed within two or four weeks and are replaced with a cancellous bone graft [22, 23]. In this study, we used cement rods instead of cement beads. We found that the cement rods are more easily made and convenient for gradual pull-out from the wound later.

Ilizarov external fixation allows reconstruction of segmental defects and difficult infected nonunions [24]. As the patients with IRI and bone defects longer than 1 cm were not included in this study, we did not routinely use this technique. But this technique is useful as a salvage of the failures of other methods. The technique is labor-intensive and requires an extended period of treatment with the device, which may last from several months to a couple of years.

Free vascularized muscle transfers improve the local biological environment by bringing in a blood supply important for host defense mechanisms, antibiotic delivery, and osseous and soft-tissue healing. But they are time consuming and require microsurgery expertise; we usually use them for large size and deep defects. In recent years, perforator flaps become more popular due to their aesthetic results and less donor site morbidity [25–28]. We chose pedicled fibular perforator flaps for small size defects, free anterolateral thigh perforator flap, free DIEP flap, and free thoracodorsal artery perforator flap for large and superficial wounds. Whenever possible, we prefer the sequent or reverse sural flap [29, 30] or local gastrocnemius flap for soft tissue reconstructions because the techniques are simple and fast. With proper preoperative evaluation and appropriate flap selection, the incidence of postoperative complications could be maximally reduced.

## 5. Conclusions

New appearing advances in the surgical region were used and combined for the treating of IRI in the tibia. These advances include the more effective external fixators for bone fixation, the self-made vancomycin cement bar for persisting local antibiotics, the vacuum sealing system for irrigation, and the perforator flaps for more choices of soft tissue reconstruction. The combination of these techniques seems to produce promising outcomes.

## Figures and Tables

**Table 1:** The treatment results of 217 cases of IRI in the tibia.

Group	Cases	Osteomyelitis healed (%)	Bone union
IRI and soft tissue defects	152	131 (86.2%)	147 (96.7%)
Simple IRI	65	57 (87.7%)	64 (98.5%)

Total	217	188 (86.6%)	211 (97.2%)
